# Correlation between quantitative perfusion histogram parameters of DCE-MRI and PTEN, P-Akt and m-TOR in different pathological types of lung cancer

**DOI:** 10.1186/s12880-021-00604-5

**Published:** 2021-04-17

**Authors:** Bingqian Zhang, Zhenhua Zhao, Ya’nan Huang, Haijia Mao, Mingyue Zou, Cheng Wang, Guangmao Yu, Minming Zhang

**Affiliations:** 1 Department of Radiology, Shaoxing People’s Hospital (Shaoxing Hospital, Zhejiang University School), No. 568, North Zhongxing Road, Yuecheng District, Shaoxing City, 312000 Zhejiang Province China; 2 Department of Pathology, Shaoxing People’s Hospital (Shaoxing Hospital, Zhejiang University School), Shaoxing, 312000 China; 3 Cardiothoracic Surgery, Shaoxing People’s Hospital (Shaoxing Hospital, Zhejiang University School), Shaoxing, 312000 China; 4 Department of Radiology, The Second Affiliated Hospital of Zhejiang University, Hangzhou, 310009 China

**Keywords:** Lung cancer, Dynamic contrast-enhanced magnetic resonance imaging, Histogram, PTEN, P-Akt, M-TOR

## Abstract

**Background:**

To explore if the quantitative perfusion histogram parameters of dynamic contrast-enhanced magnetic resonance imaging (DCE-MRI) correlates with the expression of PTEN, P-Akt and m-TOR protein in lung cancer.

**Methods:**

Thirty‐three patients with 33 lesions who had been diagnosed with lung cancer were enrolled in this study. They were divided into three groups: squamous cell carcinoma (SCC, 15 cases), adenocarcinoma (AC, 12 cases) and small cell lung cancer (SCLC, 6 cases). Preoperative imaging (conventional imaging and DCE-MRI) was performed on all patients. The Exchange model was used to measure the phar- macokinetic parameters, including K^trans^, V_p_, K_ep_, V_e_ and F_p_, and then the histogram parameters meanvalue, skewness, kurtosis, uniformity, energy, entropy, quantile of above five parameters were analyzed. The expression of PTEN, P-Akt and m-TOR were assessed by immunohistochemistry. Spearman correlation analysis was used to compare the correlation between the quantitative perfusion histogram parameters and the expression of PTEN, P-Akt and m-TOR in different pathological subtypes of lung cancer.

**Results:**

The expression of m-TOR (*P* = 0.013) and P-Akt (*P* = 0.002) in AC was significantly higher than those in SCC. V_p_ (uniformity) in SCC group, K^trans^ (uniformity), V_e_ (kurtosis, Q10, Q25) in AC group, F_p_ (skewness, kurtosis, energy), V_e_ (Q75, Q90, Q95) in SCLC group was positively correlated with PTEN, and F_p_ (entropy) in the SCLC group was negatively correlated with PTEN (*P* < 0.05); K_ep_ (Q5, Q10) in the SCLC group was positively correlated with P-Akt, and K_ep_ (energy) in the SCLC group was negatively correlated with P-Akt (*P* < 0.05); K_ep_ (Q5) in SCC group and V_p_ (meanvalue, Q75, Q90, Q95) in SCLC group was positively correlated with m-TOR, and V_e_ (meanvalue) in SCC group was negatively correlated with m-TOR (*P* < 0.05).

**Conclusions:**

The quantitative perfusion histogram parameters of DCE-MRI was correlated with the expression of PTEN, P-Akt and m-TOR in different pathological types of lung cancer, which may be used to indirectly evaluate the activation status of PI3K/Akt/mTOR signal pathway gene in lung cancer, and provide important reference for clinical treatment.

## Background

Lung cancer is one of the common malignant tumors that seriously threaten the health and life of the population. It is the malignant tumor with the highest morbidity and mortality [1]. Activation of oncogenes and the deletion and mutation of tumor suppressor genes lead to impediments in cell cycle regulation and imbalance of apoptosis regulation, which are the most fundamental causes of tumorigenesis [2]. Phosphatidylinositol 3 kinase (PI3K)/protein kinase B (AKT)/mammalian target of rapamycin (mTOR) signaling pathway plays an important role in the genesis and development of many kinds of tumors. The signaling pathways through PI3K and AKT gene mutation and amplification, oncogene receptor activation, gene expression reduction of phosphatase and tensin homolog (PTEN), and regulation of vascular endothelial growth factor (VEGF) expression and other mechanisms to promote tumor cell growth and angiogenesis [3–5]. Studies have shown that PTEN is a tumor suppressor gene with dual specific phosphatase activity, and its gene mutation and loss of expression can cause the PI3K/AKT/mTOR signaling pathway to be activated, leading to abnormal cell proliferation, infiltration and metastasis [6]. P-Akt is the activation state of AKT and is a key signal transduction factor that plays a role in promoting cancer on this signal pathway. m-TOR is the most important downstream signaling molecule of PI3K/AKT [7]. Therefore, the loss of PTEN and the abnormal activation of P-Akt and mTOR is related to tumorigenesis.

At present, Western blot and immunohistochemistry (IHC) are the main methods to detect PTEN, P-Akt, and m-TOR. However, the acquisition of tissue samples requires invasive operations such as surgery or needle biopsy, and dynamic and repeated observations cannot be performed. In addition, due to the heterogeneous tumor growth, local specimens usually cannot reflect the entire tumor. In view of the limitations of the above methods, it is clinically hoped that a non-invasive and reproducible method can be used to predict the key proteins of the carcinogenic pathway. The histogram analysis of DCE-MRI perfusion parameters can evaluate the microvascular environment of the tumor based on the gray-scale intensity information of the lesion and describe the heterogeneity of the lesion [8–10]. Therefore, the purpose of this study is to investigate the correlation between the DCE-MRI histogram parameters and PTEN, P-Akt, and m-TOR of lung cancer, so as to provide a basis for non-invasive prediction of PI3K/Akt/mTOR pathway activation in lung cancer tissue by quantitative perfusion histogram parameters of DCE-MRI.

## Methods

### Patients

The data of patients with chest MRI examinations in Shaoxing people’s Hospital from January 2017 to April 2018 were retrospectively collected. Finally, thirty‐three patients with lung cancer were enrolled for this study based on the following: Inclusion criteria: (1) all patients were diagnosed as lung cancer after surgery or biopsy; (2) the diameter of lung lesions was larger than 2 cm; (3) no biopsy, chemoradiotherapy, and drug treatment were performed before MR examination; Exclusion criteria: (1) the interval between operation or biopsy and MR examination time was more than two weeks; (3) the image scanning quality was poor or the tumor boundary could not be clearly delineated. This study was approved by the ethics committee of Shaoxing people’s hospital.

### DCE-MRI protocols

The patient was supine and the Siemens Verio 3.0T MRI scanner was used to scan the body with a twelve-element chest phased-array body coil. Routine plain scan: T2WI coronal, T1WI transverse and sagittal; T2WI transverse and sagittal; then multiphase dynamic contrast (DCE-MRI) scans. Dynamic enhancement was performed using a 3D VIBE T1-weighted dynamic perfusion sequence scan, with all sequences using free breathing. Multi-flip angle scan before dynamic enhanced scan, scan parameters: TR 3.25 ms, TE 1.17 ms, Flip Angle: 5°, 10°, 15°, field of view 350 mm × 282 mm, matrix 162 × 288, layer thickness 5 mm, The number of layers was 30, and the time resolution of each period was 6.5 s. Dynamic enhanced scanning sequence parameters: Flip Angle 10°, scanning 35 phases, the remaining parameters were the same as above, the total time of multi-flip angle scanning and dynamic enhanced scanning was 247 s. When the dynamic enhanced scan was in phase 3, a high-pressure syringe was used to inject the contrast agent gadolinium diamine (Omniscan, Ge Healthcare) through the median cubital vein. The injection dose was 0.1 mmol/kg, the injection speed was 3.0 ml/s, followed by a 20 mL saline flush.

### MR image analysis

**Fig. 1:**
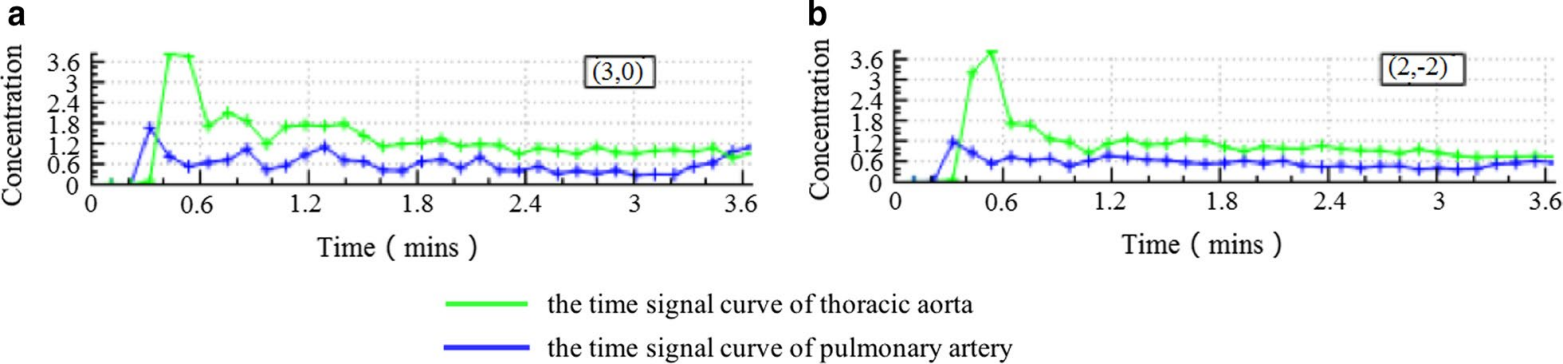
3D non rigid correction technology corrects the motion artifact of the scanning image for patients with lung cancer DCE-MRI. **a** Before registration, the time signal curve of pulmonary artery (blue line) and the time signal curve of thoracic aorta (green line) are poorly fitting; **b** after registration, the time signal curve of bronchial artery and pulmonary artery fitting is improved, and the curve is smoother

**Fig. 2:**
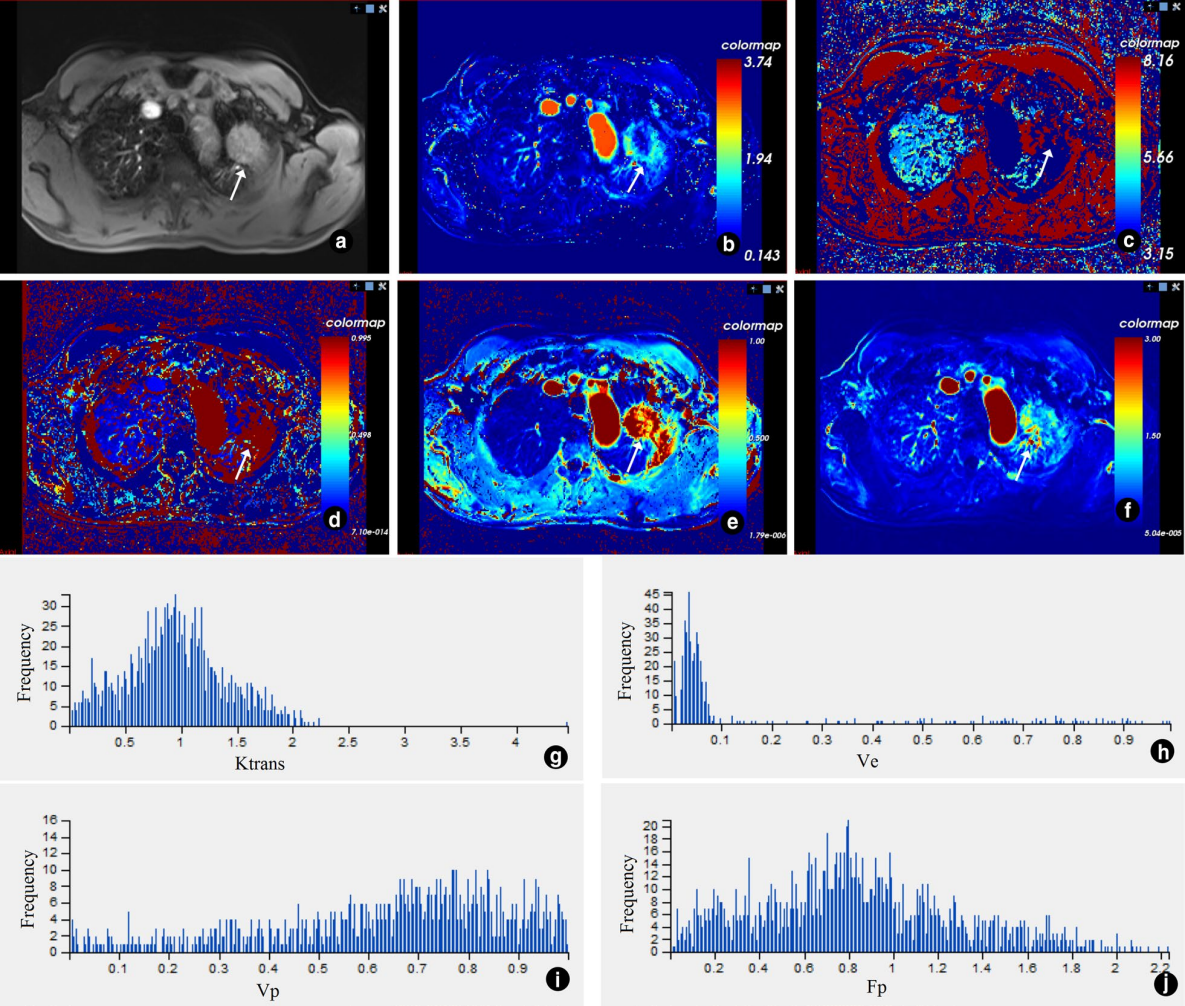
The 3D ROI image, pseudo color map and histogram of quantitative perfusion parameters of the adenocarcinoma in the upper left lung, and the white arrow points to the lesion. **a** Single slice ROI of lesion; **b** 3D ROI of lesion; **c** pseudo color map of K^trans^; **d** pseudo color map of V_e_; **e** pseudo color map of V_p_; **f** pseudo color map of F_p_; **g** histogram of K^trans^ (homogeneity = 0.621; skewness = 0.942; kurtosis = 3.827; entropy = 6.758; energy = 0.011); **h** histogram of V_e_ (homogeneity = 0.548; skewness = − 1.571; kurtosis = 0.496; entropy = 4.890; energy = 0.043; **i** histogram of V_p_ (homogeneity = 0.668; skewness = − 1.318; kurtosis = 0.960; entropy = 7.538; energy = 0.006); **j** histogram of F_p_ (homogeneity = 0.508; skewness = 0.201; kurtosis = − 0.401; entropy = 7.589; energy = 0.006)

The original DCE data after scanning was imported into Omni.Kinetics (GE Healthcare, China) software. The 3D correction technology of free breathing (no rigid medical image registration algorithm) was used to correct the motion artifacts of dynamic enhancement sequence and check the fitting state of time intensity curve (TIC) before and after registration and check the registration effect. The curve was linear and smooth, which indicated that the fitting was good (Fig. 1). Multi-flip angles of 5°, 10°, 15° and corrected dynamic enhancement sequence scan were processed by the hemodynamic software Omni.Kinetics, and the perfusion parameters were calculated by the Exchange model of bronchial artery and pulmonary artery double blood supply. Avoiding cystic change, necrosis and normal lung tissue to sketch the region of interest (ROI), 3–5 layers above and below the largest tumor layer were integrated into a 3D ROI for quantitative analysis and calculation. Five quantitative parameters were obtained: K^trans^ (transfer constant), V_p_ (fractional volume of plasma), K_ep_ (rate constant of backflux from extravascular extracellular space [EES] to plasma), V_e_ (fractional volume of EES) and F_p_ (tissue plasma perfusion). Histogram analysis of each perfusion parameter includes the following contents: meanvalue, skewness, kurtosis, uniformity, energy, entropy, Q10, Q25, Q75, Q90, Q95 (Fig. 2). The above parameters were also analyzed by Omni.Kinetics. Data processing was measured three times by two senior radiologists with more than five years’ experience in chest imaging diagnosis, and the average value was taken.

### Immunohistochemical evaluation of PTEN, P-Akt and m-TOR

PTEN, P-Akt and m-TOR were assessed by immunohistochemistry (IHC) using paraffin‐embedded tissue samples which were obtained from surgery or biopsy. The process was performed according to the IHC protocol. Briefly, all sections were deparaffinized and rehydrated, and antigen retrieval was performed before immunohistochemical staining. Non‐specific binding sites were blocked by serum blocking solution at 37 °C for 10 min (Dako company). The sections were stained with monoclonal mouse anti‐human PTEN antibody (1:600, M3627, Dako, Beijing, China) [11], monoclonal rabbit anti-human P-Akt antibody (1:200, T308, Abcam, Shanghai, China) [12], or monoclonal rabbit anti-human mTOR antibody (1:400, 2983, CST, Shanghai, China) [12], and 4 °C overnight. The specimens were stained with secondary antibody and were then incubated at 37 °C for 10 min. DBA staining, rinsing, mild counterstaining of hematoxylin. After dehydration, transparency and mounting, the slides were visualized using a microscope. The immunostaining results of specific antibodies were measured semi-quantitatively by immunoreactive score (IRS) method [13]. The specific calculation method of IRS is as follows: Staining intensity (SI) classification: 0, no staining; 1, light yellow; 2, brown yellow, 3, dark brown; percentage of stained cells (PP): 0, no staining; 1, staining in < 10% of tumor cells; 2, staining in 10–50% of cells; 3, staining in 50–80% of cells; 4, staining in > 80% of cells. IRS = SI × PP. A pathologist with more than 5 years of experience will perform the interpretation of the results.

### Statistical analysis

Shapiro–Wilk test was used to check normality assumption for quantitative variables. The classification variables were analyzed by Fisher exact test. One-way analysis of variance (AVONA) and Least Significant Difference (LSD) were used to compare the expression of PTEN, m-TOR and P-Akt protein among different pathological groups of lung cancer, Kruskal Walls test were used to compare the perfusion histogram parameters among three groups of lung cancer; Nonparametric correlation analysis (Spearman test) was used to analyze the correlation between PTEN, m-TOR and P-Akt and DCE-MRI quantitative perfusion histogram parameters measured by the Exchange model. Statistical analyses were performed using the SPSS (version. 25.0, Chicago, IL, USA). For all the above-mentioned analyses, *P* < 0.05 was considered statistically significant.

## Result

### Demographic of patients with lung cancer

Table 1 summarizes the demographic of the three groups of patients with lung cancer subtypes. There were 15 cases of squamous cell carcinoma (SCC), 12 cases of adenocarcinoma (AC) and 6 cases of small cell lung cancer (SCLC). The median age, mean age ± standard deviation (SD) and age range were 66, 66.79 ± 9.24 and 50–85 years, respectively. There was no significant difference in sex, age, BMI and tumor maximum diameter among the three groups (*P* = 0.059; F = 0.830, *P* = 0.446; F = 1.459, *P* = 0.250; F = 0.593, *P* = 0.559).

**Table 1:** Demographic of patients with lung cancer

Characteristics	SCC (n = 15)	AC (n = 12)	SCLC (n = 6)	total	F value	*P* value
Gender						0.059
Male	15 (100.0%)	9 (75.0%)	4 (66.7%)	28 (84.8%)		
Female	0 (0%)	3 (25.0%)	2 (33.3%)	5 (15.2%)		
Age (years, x ± s)	68.3 ± 9.9	67.0 ± 10.2	62.5 ± 3.6		0.830	0.446
Age range	51–85	50–84	58–67			
BMI (Kg/m^2^)	21.2 ± 2.6	20.9 ± 3.5	23.4 ± 2.8		1.459	0.250
Tumor maximum						
Diameter (mm)	63.4 ± 28.9	55.5 ± 26.9	51.0 ± 11.7		0.593	0.559

### Expression of PTEN, P-Akt and m-TOR in different types of lung cancer

**Fig. 3:**
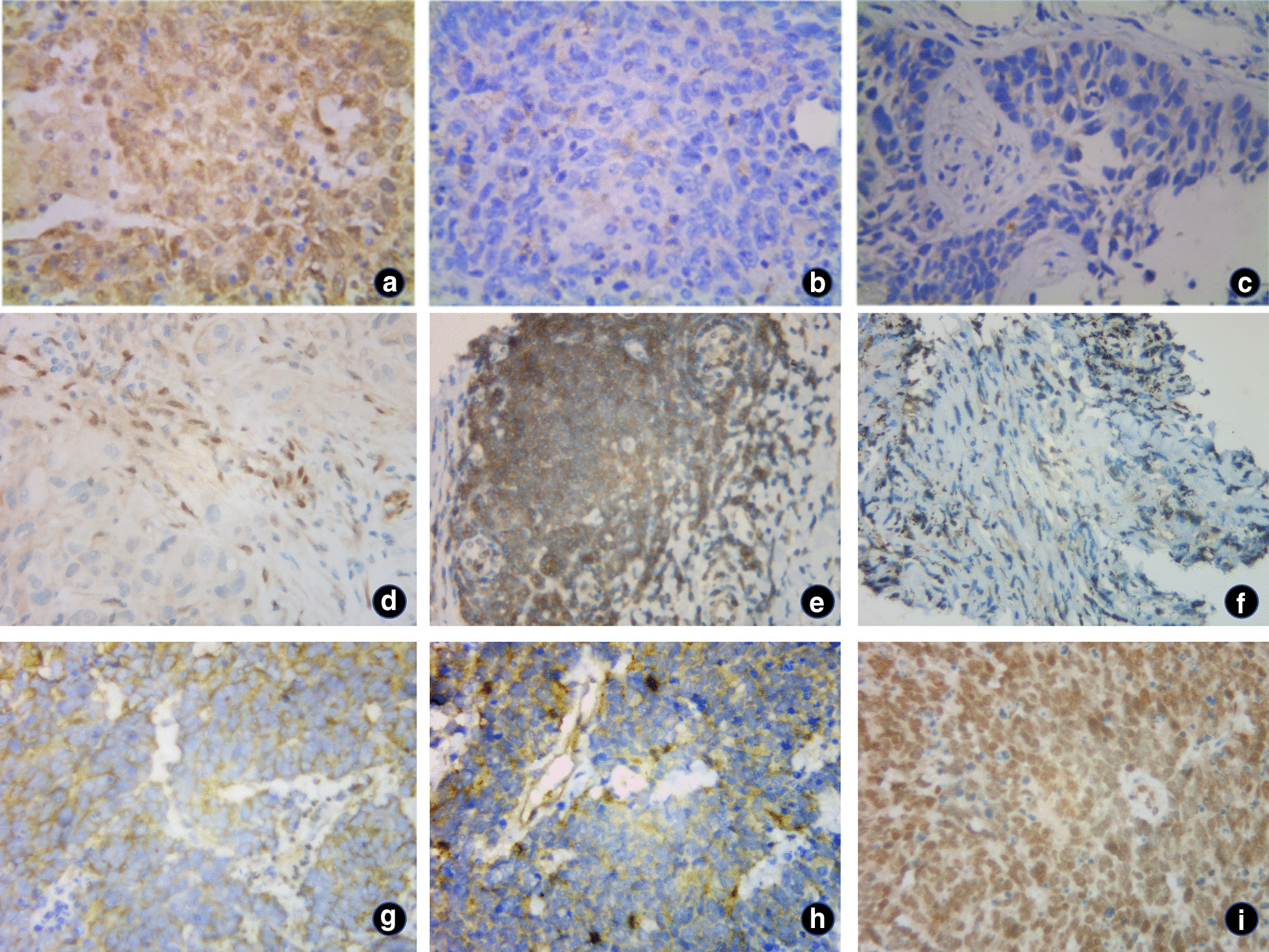
IHC images. **a**–**c** IHC images of a case of SCC; **a** PTEN, IRS: 8; **b** P-Akt, IRS: 1; **c** m-TOR, IRS: 2. **d**–**f** IHC images of a case of AC; **d** PTEN, IRS: 2; **e** P-Akt, IRS: 12; **f** m-TOR, IRS: 8. **g**–**i** IHC images of a case of SCLC; **g** PTEN, IRS: 8; **h** P-Akt, IRS: 6; **i** m-TOR, IRS: 1. (magnification: 10 × 40)

**Fig. 4:**
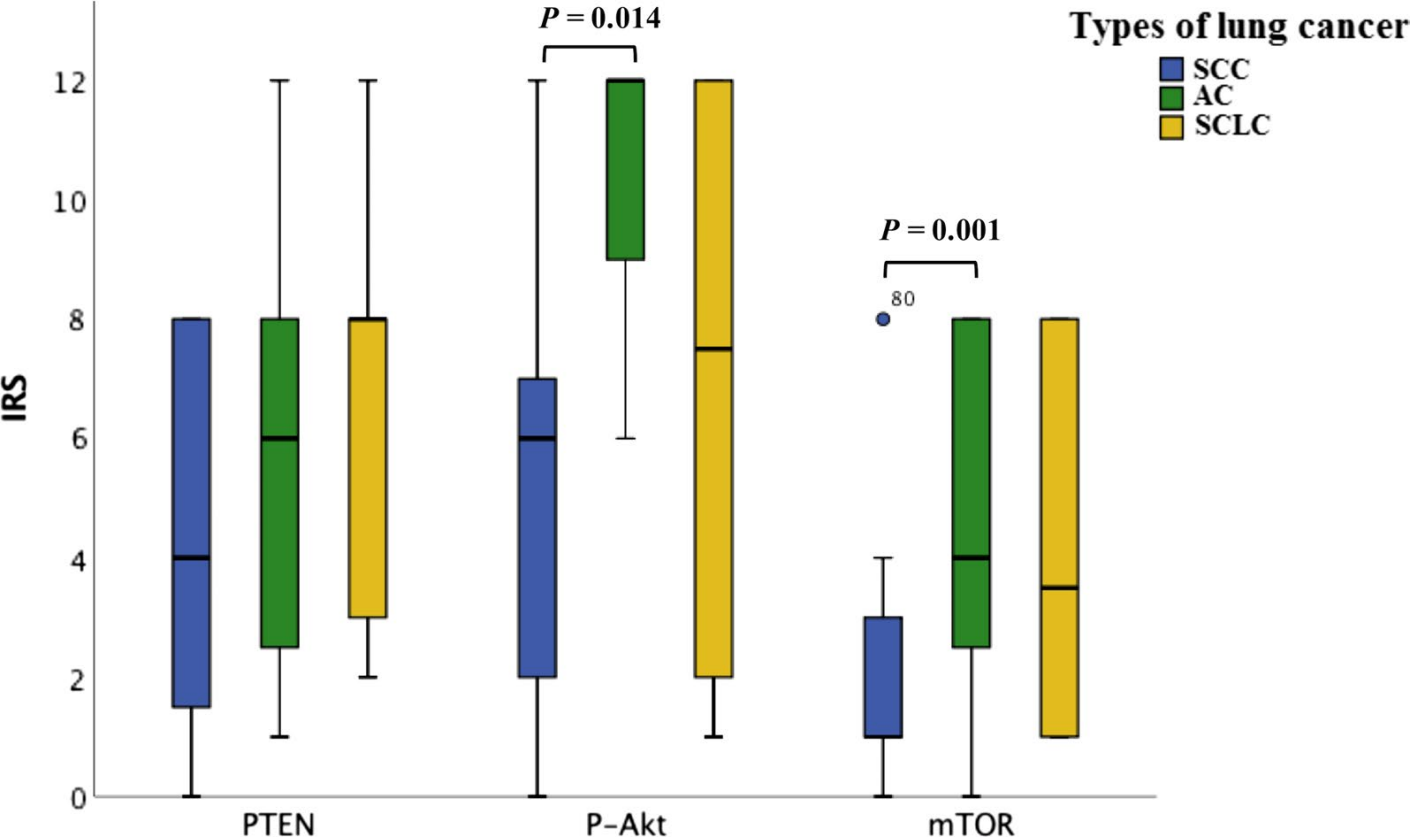
The difference of PTEN, m-TOR and P-Akt among three types of lung cancer. There was no significant difference in PTEN expression among the three groups; The expression of P-Akt in AC was significantly higher than that in SCC (*P* = 0.014); The expression of m-TOR in AC was significantly higher than that in SCC (*P* = 0.001)

PTEN, m-TOR and P-Akt expression were identified by IHC (Fig. 3). As shown in Fig. 4, PTEN expression in SCC was lower than that in the other two groups, but not statistically significant(*P* > 0.05). The expression of m-TOR and P-Akt in AC was higher than that in the other two groups, and significantly higher than that in SCC patients (*P* = 0.014; *P* = 0.001).

### Correlations of perfusion histogram parameters with PTEN, P-Akt and m-TOR expression

**Fig. 5:**
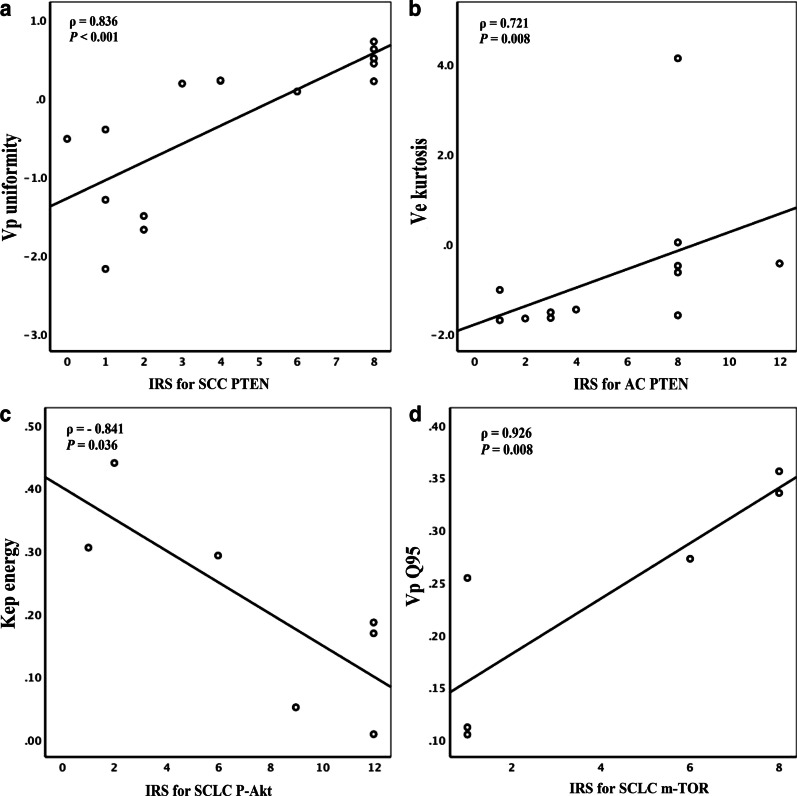
Scatter diagram between PTEN, P-Akt, m-TOR and DCE-MRI perfusion histogram parameters of lung cancer. **a** Scatter diagram of uniformity of V_p_ in SCC and PTEN (ρ = 0.836, *P* < 0.001); **b** Scatter diagram of kurtosis of V_e_ in AC and PTEN (ρ = 0.721, *P* = 0.008); **c** Scatter diagram of energy of K_ep_ in SCLC and P-Akt (ρ = − 0.841, *P* = 0.036); **d** Scatter diagram of quantile95 of V_p_ in SCLC and m-TOR (ρ = 0.926, *P* = 0.008)

Table 2, 3 and 4 and Fig. 5 summarizes the perfusion histogram parameters significantly related to PTEN, P-Akt and m-TOR expression. The remaining perfusion histogram parameters are not significantly related to PTEN, P-Akt and m-TOR expression.

**Table 2:** Correlation between perfusion histogram parameters and PTEN expression in three types of lung cancer

Group	Kinetic parameter	Histogram metrics	*ρ* value	*P* value
SCC	V_p_	Uniformity	0.836	< 0.001
AC	K^trans^	Uniformity	0.633	0.027
	V_e_	Kurtosis	0.721	0.008
		Q10	0.590	0.044
		Q25	0.611	0.035
SCLC	F_p_	Skewness	0.941	0.005
		Kurtosis	0.941	0.005
		Energy	0.941	0.005
		Entropy	− 0.941	0.005
	V_e_	Q75	0.820	0.046
		Q90	0.941	0.005
		Q95	0.820	0.046

**Table 3:** Correlation between perfusion histogram parameters and P-Akt expression in three types of lung cancer

Group	Kinetic parameter	Histogram metrics	*ρ* value	*P* value
SCLC	K_ep_	Energy	− 0.841	0.036
		Q5	0.841	0.036
		Q10	0.841	0.036

**Table 4:** Correlation between perfusion histogram parameters and m-TOR expression in three types of lung cancer

Group	Kinetic parameter	Histogram metrics	*ρ* value	*P* value
SCC	K_ep_	Q5	0.760	0.001
	V_e_	Meanvalue	− 0.619	0.014
SCLC	V_p_	Meanvalue	0.926	0.008
		Q75	0.926	0.008
		Q90	0.926	0.008
		Q95	0.926	0.008

PTEN was positively correlated with V_p_ (uniformity) in SCC (ρ = 0.836, *P* < 0.001), K^trans^ (uniformity) in AC (ρ = 0.633, *P* = 0.027), V_e_ (kurtosis, Q10, Q25) in AC (ρ = 0.721, *P* = 0.008; ρ = 0.590, *P* = 0.044; ρ = 0.611, *P* = 0.035), F_p_ (skewness, kurtosis, energy) in SCLC (ρ = 0.941, *P* = 0.005)and V_e_ (Q75, Q90, Q95) in SCLC (ρ = 0.820, *P* = 0.046; ρ = 0.942, *P* = 0.005; ρ = 0.820, *P* = 0.046). While PTEN was negatively correlated with F_p_ (entropy) in the SCLC (ρ = − 0.941, *P* = 0.005).

P-Akt was positively correlated with K_ep_ (Q5, Q10) in the SCLC (ρ = 0.841, *P* = 0.036). While P-Akt was negatively correlated with K_ep_ (energy) in the SCLC (ρ = − 0.841, *P* = 0.036);

m-TOR was positively correlated with K_ep_ (Q5) in SCC (ρ = 0.760, *P* = 0.001) and V_p_ (meanvalue, Q75, Q90, Q95) in SCLC (ρ = 0.926, *P* = 0.008). While m-TOR was negatively correlated with V_e_ (meanvalue) in SCC (ρ = − 0.619, *P* = 0.014).

## Discussion

Under physiological conditions, PI3K/Akt/mTOR pathway plays a role in cell survival, proliferation and angiogenesis. The disorder of this pathway will promote the occurrence and development of tumor. Currently, immunohistochemistry and Western blotting are mainly used to detect the expression of PTEN, P-Akt and m-TOR. We observed P-Akt and m-TOR were more frequently expressed in adenocarcinoma histology than in squamous cell carcinoma, and PTEN was not significantly correlated with pathological type (Fig. 4). It may be suggested that the expression of m-TOR and P-Akt is inconsistent in the development of AC and SCC. Previously, Oh MH et al. reported that there was more frequent expression of P-Akt and m-TOR in AC compare with SCC [14], which is consistent with our results. However, the acquisition of tissue samples requires invasive operations, and because of the heterogeneity of tumor growth, local specimens usually cannot reflect the entire tumor. In view of the limitations of the above methods, it is clinically desirable to use a non-invasive method that can reflect tumor heterogeneity to evaluate tumor characteristics. DCE‐MRI has been used as an imaging biomarker to evaluate of tumor heterogeneity, chemotherapy response and prognosis of lung cancer [15, 16]. Considering the heterogeneity of tumor tissue and microvascular distribution, the histogram analysis of perfusion parameters has been applied in various fields of tumor research [17]. Commonly used histogram parameters include: meanvalue, quantile, skewness, kurtosis, uniformity, entropy, energy, etc. Based on the clinical utility of PI3K/Akt/mTOR pathway related proteins, the current study was designed to examine the association between DCE-MRI quantitative perfusion histogram parameters and tumor biomarkers.

PTEN is the first tumor suppressor gene with phosphatase activity. The reduced expression of PTEN in tumor cells will continuously stimulate PI3K Signal Pathway, thereby increasing cell proliferation, invasion and metastasis [18, 19]. In NSCLC, PTEN loss is associated with poor clinical outcomes, and resistance to many anticancer drugs, including gefitinib [20, 21]. In this study, V_p_ (uniformity) in SCC group, K^trans^ (uniformity) in AC group was positively correlated with PTEN. K^trans^ and V_p_ are closely related to the intravascular plasma flow and vascular wall permeability. Because the tumor grows too fast, it often causes the tumor heterogeneity to be remarkable, namely the uniformity reduces in histogram. The previous study showed that tumor progression appears to be linked to expansion of histograms to the right (decreased skewness) and peak broadening with decreased height (decreased kurtosis) [22, 23]. In the current study, V_e_ (kurtosis) in AC group, F_p_ (skewness, kurtosis) in SCLC group was positively correlated with PTEN. The results are in accordance with previous studies. In SCC, PTEN was positively correlated with F_p_ (energy), while negatively correlated with F_p_ (entropy). Entropy represents the complexity of texture in image, which is opposite to energy. With the absence of PTEN, the F_p_ parameter histogram shows a decrease in energy and an increase in entropy value, suggesting that the internal texture features of the tumor are complex. Meng et al. [24] found that as the malignancy of cervical cancer decreases, the ADC entropy value decreases, proving that the entropy value can reflect tumor heterogeneity by reflecting the complexity of the internal texture of the lesion. V_e_ reflects the size of extracellular space. PTEN loss activates PI3K/Akt/mTOR signaling pathway, tumor proliferation is active, and EES is decreased (decreased V_e_). In this study, PTEN has no correlation with V_e_ (meanvalue), but has positive correlation with V_e_ (Q10, Q 25) in AC group and V_e_ (Q75, Q90, Q95) in SCLC group. It may be due to the spatial heterogeneity of lung cancer tissue, and the quantile describe the gray distribution of each pixel in the lesion, so the V_e_ quantile may better reflect the micro physiological structure of tumor tissue.

P-Akt is an important signal transducer in PI3K/Akt/mTOR signaling pathway. Deletion and inactivation of PTEN gene and overexpression of PI3K gene can stimulate Akt activation, and promote tumor growth, invasion and metastasis through a variety of downstream effectors (including mTOR). K_ep_ describes the rate constant of backflux from EES to plasma, which is closely related to the permeability of blood vessel wall. In this study, K_ep_ (Q5, Q10) was positively correlated with P-Akt, and K_ep_ (energy) was negatively correlated with P-Akt, indicating that the quantile and energy of K_ep_ can reflect the expression of P-Akt by evaluate the heterogeneity and microvascular permeability of lung cancer tissues. A possible explanation is that PI3K/Akt pathway up-regulates the expression of hypoxia-inducible factor-1 (HIF-1), thereby activates vascular endothelial growth factor (VEGF) expression to mediate angiogenesis [25].

As the most important downstream signal molecule of PI3K/Akt, mTOR is activated by Akt phosphorylation, and the expression of mTOR is abnormally increased in tumor progression. In this study, we found that K^trans^ (Q5) in SCC was positively correlated with m-TOR, and V_e_ (mean) in SCC was negatively correlated with m-TOR in SCC group. The activation of m-TOR promotes tumor proliferation. V_e_ is related to the size of extracellular space. The more active the tumor proliferation, the smaller V_e_. With the growth of tumor, the angiogenesis of tumor increases, which shows the decrease of V_e_ and the increase of K^trans^ in histogram. V_p_ reflects the amount of tissue perfusion. The positive correlation between V_p_ (meanvalue, Q75, Q90, Q95) and mTOR in SCLC also indicated that the activation of downstream signal molecules of this signal pathway increased tumor blood supply.

Our research has several limitations. First of all, the motion artifacts of pulmonary DCE-MRI were common due to respiration. However, we used 3D non rigid correction technology to reduce the impact of motion artifacts on the study. Secondly, due to the limitations of MRI in the use of lung, the sample size of this study was small, especially in small cell lung cancer, which needed to be expanded for further study. Third, the spatial distribution of perfusion parameters is uneven, especially in the peritumoral region, so it is difficult for histopathological samples to accurately match the corresponding ROI delineation regions.

## Conclusions

In conclusion, DCE-MRI quantitative perfusion histogram can be used as a noninvasive, in vivo and reproducible method to indirectly evaluate the activation of PI3K/Akt/mTOR signal pathway gene in lung cancer, and provide a way for MRI to evaluate tumor heterogeneity at the molecular level, which is of great significance to clinical practice.

## Data Availability

The datasets generated and/or analysed during the current study are not publicly available due [We will continue to expand the sample size for follow-up research] but are available from the corresponding author on reasonable request.

## Abbreviations

DCE-MRI: Dynamic contrast-enhanced magnetic resonance imaging
SCC: Squamous cell carcinoma
AC: Adenocarcinoma
SCLC: Small cell lung cancer
AKT: Protein kinase B
mTOR: Mammalian target of rapamycin
PTEN: Phosphatase and tensin homolog
ROI: Region of interest
K^trans^: Transfer constant
V_p_: Fractional volume of plasma
K_ep_: Rate constant of backflux from extravascular extracellular space [EES] to plasma
V_e_: Fractional volume of EES
F_p_: Tissue plasma perfusion
